# Supplementary material for: Bayesian refinement of protein structures and ensembles against SAXS data using molecular dynamics
Source: PLoS Comput Biol. 2017 Oct 18;13(10):e1005800. doi: 10.1371/journal.pcbi.1005800 (PMC5662244; doi:10.1371/journal.pcbi.1005800)
Supplement: S1 Archive — (BZ2) [file pcbi.1005800.s011.tar.bz2 › gromacs_4.62_bayesian_saxs_refi/share/html/online/genpr.html]

genpr


| genpr Main Table of Contents |

**VERSION 4.6  
Sat 19 Jan 2013**

---

### Description

genpr produces an include file for a topology containing
a list of atom numbers and three force constants for the
X, Y and Z direction. A single isotropic force constant may
be given on the command line instead of three components.

WARNING: genpr only works for the first molecule.
Position restraints are interactions within molecules, therefore
they should be included within the correct [ moleculetype ]
block in the topology. Since the atom numbers in every moleculetype
in the topology start at 1 and the numbers in the input file for
genpr number consecutively from 1, genpr will only produce a useful
file for the first molecule.

The -of option produces an index file that can be used for
freezing atoms. In this case the input file must be a pdb file.

### Files

| option | filename | type | description |
| --- | --- | --- | --- |
| **-f** | conf.gro | Input | Generic structure: gro g96 pdb tpr tpb tpa xml |
| **-n** | index.ndx | Input, Opt. | Index file |
| **-o** | posre.itp | Output | Include file for topology |
| **-of** | freeze.ndx | Output, Opt. | Index file |

### Other options

| option | type | default | description |
| --- | --- | --- | --- |
| **-[no]h** | bool | no | Print help info and quit || **-nice** | int | 0 | Set the nicelevel || **-fc** | vector | 1000 1000 1000 | force constants (kJ mol-1 nm-2) || **-freeze** | real | 0 | if the -of option or this one is given an index file will be written containing atom numbers of all atoms that have a B-factor less than the level given here |

---

http://www.gromacs.org  
gromacs@gromacs.org
